# Correlations between cytokines produced by T cells and clinical-virological characteristics in untreated chronic hepatitis B patients

**DOI:** 10.1186/s12879-019-3853-2

**Published:** 2019-03-04

**Authors:** Yurong Gu, Yifan Lian, Lin Gu, Lubiao Chen, Xiaoyan Li, Liang Zhou, Yanlin Huang, Jialiang Wang, Yuehua Huang

**Affiliations:** 1 Department of Infectious Diseases, The Third Affiliated Hospital of Sun Yat-sen University, 600 Tian He Rd., Guangzhou, 510630 China; 2 Guangdong Provincial Key Laboratory of Liver Disease Research, The Third Affiliated Hospital of Sun Yat-sen University, Guangzhou, China

**Keywords:** Chronic hepatitis B (CHB), Cytokines, Linear regression analysis

## Abstract

**Background:**

Hepatitis B virus (HBV) replicates non-cytopathically in the hepatocytes and HBV-related diseases are caused by immune-mediated inflammatory events. This study aimed to identify the relationship between clinical-virological characteristics and immunity in untreated chronic hepatitis B (CHB) patients.

**Methods:**

A total of 209 CHB patients were categorized into immune tolerant (IT, *n* = 17), inactive carrier (IC, *n* = 20), immune active (IA, *n* = 120), and gray zone (GZ, *n* = 72) phases. The quantitative hepatitis B surface antigen (qHBsAg), hepatitis B e antigen (HBeAg), anti-HBeAg (HBeAb), HBV genotype, viral mutant and frequencies of interleukin (IL)-4, IL-17, IL-10 and interferon-gamma (IFN-γ) produced by CD4^+^ and CD8^+^ T cells were tested. We also correlated these cytokines with clinical-virological characteristics using a linear regression model.

**Results:**

CD8^+^ T cells frequency were significantly decreased in IT patients. Levels of CD4^+^ T cells IL-4^+^ or IL-10^+^ were strongly negatively associated with qHBsAg titers. The frequency of IFN-γ produced by CD4^+^ and CD8^+^ T cells showed significant positive association with age and alanine aminotransferase (ALT) level, while that had negative association with qHBsAg titers. Additionally, the ratios of mutations in the HBV precore (PC) stop codon and basal core promoter (BCP) and the combined mutations were 32.5, 27.2, and 11.3%, respectively. The frequency of CD4^+^ T cells IL-17^+^ was higher in patients with a PC mutation than that in patients carrying a wild-type sequence. Finally, little associations among T cell derived IL-4, IL-10, IL-17, and IFN-γ was observed in the current untreated CHB cohort.

**Conclusions:**

Several components of the immune system were correlated with HBV factors that influence an inflammatory process during CHB. Of particular relevance are the significant associations of between CD4^+^ T cells IL-4^+^ and qHBsAg level, and between CD4^+^ T cells IL-17^+^ and the presence of a mutation in PC.

## Background

Although a prophylactic vaccine and effective antiviral therapies are available, chronic hepatitis B (CHB) remains a significant global health threat, affecting more than 240 million people [1]. The Asian-Pacific region is a hyper-endemic area of chronic hepatitis, and a majority of hepatitis B virus (HBV) infections eventually progress to end-stage liver diseases including cirrhosis, as well as to hepatocellular carcinoma (HCC) [2].

HBV belongs to *Hepadnaviridae*, a family of enveloped viruses with an incomplete double-stranded DNA genome of 3.2 kb [3]. The quantitative level of serum hepatitis B surface antigen (HBsAg) has been suggested to serve as an indicator of a response to antiviral treatment [4, 5]. However, the serum level of HBsAg is dynamic, and its correlation with the level of serum HBV DNA appears to change in different phases of the natural history of chronic hepatitis B [6, 7]. According to current practice guidelines, the disease status of CHB patients can be grouped into four phases, as indicated by the level of hepatitis B e antigen (HBeAg), serum ALT and HBV DNA: immune tolerant (IT), immune active (IA); inactive carrier (IC); gray zones (GZ) [8].

In addition to HBV antigen titers and viral load, the HBV genotype and virus mutations have been well documented as risk predictors of cirrhosis and HCC in CHB patients [9–12]. Based on sequence divergence > 8% for the entire HBV genome, 10 genotypes (A–J) have been identified to date [12]. HBV genotypes have distinct geographical distributions worldwide and differ with regard to clinical diseases, prognosis, and response to interferon treatment [13, 14]. In addition, mutations in different parts of the viral genome occur due to the stress from humoral and cell-mediated immunities. Core promoter mutants are not only associated with an increased risk of HCC development but also have a direct relationship with poor survival in postoperative HCC patients [15, 16]. Another common mutation, G1896A (which changes a TGG tryptophan codon to a TAG stop codon), in the precore (PC) region of the virus has been identified as being related to HBeAg seroconversion. Patients carrying this PC mutation do not express HBeAg and still suffer from severe liver disease [17, 18].

It is clear that HBV replicates non-cytopathically in the hepatocytes and that virus-related diseases are caused by chronic immune-mediated inflammatory events [19]. During the course of liver damage, adaptive immune cells are crucially involved in the pathogenesis of hepatic inflammation, with T cells playing particularly important roles in antiviral defense [20–22]. T lymphocyte activity is largely dependent on the differentiation of naïve T cells into T helper cells or effector T cell subsets via the release of cytokines [23]. It is well documented both in vitro and in vivo that antiviral T cell function is more efficient in patients in whom infection can be controlled either partially, such as for inactive HBsAg carriers with low levels of virus replication, or completely, such as for patients who achieve HBsAg loss either spontaneously or after longitudinal antiviral therapy [24, 25]. Moreover chronic inflammation alters not only the access and function of HBV-specific T cells in the liver parenchyma, but also the ability of secreted cytokines to activate antiviral mechanisms [26, 27]. Persistent exposure of T cells to HBV antigens is important for maintaining depressed T cell functionality [28].

Although previous studies have demonstrated the roles of T cell immune responses and viral factors in the progression of CHB [29], few studies have evaluated the associations of different subpopulations of T cells and their cytokines with clinical-virological factors using a logistic model adjusted for age, sex, HBV parameters, liver fibrosis and inflammation in a treatment naïve CHB cohort. Therefore, in this study, we measured T cell subsets as well as their cytokines, including CD4^+^ and CD8^+^ T cells, effector cytokines IFN-γ and interleukin (IL)-17, and Th2 cytokine IL-4 and immunomodulatory cytokine IL-10, in naïve CHB patients at different disease phases. Correlations between T cell-produced cytokines and clinical-virological characteristics was also analyzed.

## Methods

### Patients

Consecutive adult patients with CHB infection observed in the dedicated viral hepatitis clinic of The Third Affiliated Hospital of Sun Yat-sen University between July 2015 and July 2016 were recruited. Patients were excluded for the following reasons: receiving antiviral treatment (IFN-α or nucleoside analogs) within the previous 6 months; patients with human immunodeficiency virus (HIV), hepatitis C virus, or hepatitis D virus coinfection; and patients with end-stage liver insufficiency, autoimmune disorders, immunosuppressive treatment, cirrhosis, and malignancies. Written informed consent was obtained from all patients. The study was approved by the Institutional Review Board of the Sun Yat-sen University, and it conforms with the provisions of the Declaration of Helsinki.

Of the 244 individuals eligible for participation in this study, 15 were excluded because of missing data, leaving 229 patients available for analysis. The classification and denomination of CHB patients in this study was based exclusively on serological and biochemical parameters, in accordance with published international treatment guidelines, as follows: (1) IT phase - normal alanine aminotransferase (ALT) level, elevated HBV DNA, typically > 1 million IU/mL, HBeAg positivity; (2) IA phase - elevated ALT level, HBeAg positivity, HBV DNA > 20,000 IU/mL, or HBeAg negativity HBV DNA > 2000 IU/mL; (3) IC phase - normal ALT level, anti-HBsAg (HBeAb) positivity, low HBV DNA level; (4) GZ phase - ALT and HBV DNA levels do not falling into the same above traditionally characterized phases [8]. In addition, blood was also obtained from age and sex matched non-HBV infected healthy controls (*n* = 17, *p* = 0.77 and 0.98 for age and sex respectively, compared with CHB patients). Healthy controls were recruited from Physical Examination Center in our hospital. Information on the demographics (age range, sex distribution), HBV markers (HBV DNA, HBeAg, HBsAg, HBeAb, anti-HBsAg [HBsAb]), ALT, HBV genotypes, and HBV mutants (PC and basal core promoter [BCP] mutants) of CHB patients and healthy controls is listed in Table 1.

**Table 1:** Clinical-virological characteristics of patients included in the study

Characteristics	IT (*n* = 17)	IA (*n* = 120)	IC (*n* = 20)	GZ (*n* = 72)	HC (*n* = 17)	*P* value
Age, years, median (quartile)	25 (24, 26)	29 (25, 33.25)	32 (28.75, 37)	31.5 (26, 38.25)	27 (25.5, 36)	< 0.001
Gender						0.238
Male, *n* (%)	12 (70.6)	77 (64.2)	17 (85)	55 (76.4)	12 (70.6)	
Female, *n* (%)	5 (29.4)	43 (35.8)	3 (15)	17 (23.6)	5 (29.4)	
ALT, U/L, median (quartile)	20.6 (18.3, 22.4)	20.8 (19.1, 22.5)	22.3 (21.6, 23.8)	21.484 (19.8, 23.4)	16 (12.5, 22.0))	< 0.001
Fibroscan, Kpa median (quartile)	4.9 (4.2, 5.4)	5.3 (4.3, 6.5)	4.4 (4.0, 5.3)	4.8 (4.4, 5.4)	4.6 (4.0~5.1)	0.016
HBV DNA, Log IU/ml, median (quartile)	8.2 (8.2, 8.2)	7.7 (5.0, 8.2)	2.2 (1.6, 3.1)	3.3 (2.1, 4.3)		< 0.001
HBeAg status						< 0.001
Negative, *n* (%)	0 (0)	41 (34)	20 (100)	62 (86)		
Positive, *n* (%)	17 (100)	78 (65)	0 (0)	10 (14)		
Missing, *n* (%)	0 (0)	1 (1)	0 (0)	0 (0)		
HBeAb status						< 0.001
Negative, *n* (%)	17 (100)	70 (58.3)	1 (5)	13 (18.1)		
Positive, *n* (%)	0 (0)	47 (39.2)	19 (95)	59 (81.9)		
Missing, *n* (%)	0 (0)	3 (2.5)	0 (0)	0 (0)		
qHBsAg, Log IU/ml, median (quartile)	4.6 (4.5, 4.7)	4.0 (3.3, 4.7)	2.9 (2.0, 3.2)	3.2 (2.3, 3.6)		< 0.001
HBsAb status						< 0.001
Negative, *n* (%)	15 (88)	106 (88)	20 (100)	67 (93)		
Positive, *n* (%)	2 (12)	14 (12)	0 (0)	5 (7)		
HBV genotype						< 0.001
C, *n* (%)	2 (12)	30 (25)	3 (15)	15 (21)		
B, *n* (%)	12 (71)	74 (62)	8 (40)	27 (38)		
Other, *n* (%)	1 (6)	10 (8)	9 (45)	29 (40)		
Missing, *n* (%)	2 (11)	6 (5)	0 (0)	1 (1)		

HBV genotype: Other included C + D, B + D, B + C, D, and not detected

IT, immune tolerant; IA, immune active; IC, inactive carrier; GZ, gray zones; ALT, alanine aminotransferase; HBeAb, antibody to HBV e antigen; HBeAg, HBV e antigen; HBsAb: antibody to hepatitis B surface antigen; HBsAg, hepatitis B surface antigen; PC: precore; BCP: basal core promoter

### Cell-surface and cytokines staining and flow cytometry analysis

Peripheral blood mononuclear cells (PBMCs) were isolated from fresh blood samples using Ficoll density gradients according to the manufacturer’s instructions. The isolated PBMCs were stained for surface markers, fixed, permeabilized with IntraPreReagent (Beckman Coulter, Fullerton, CA), and further stained with antibodies directed against intracellular markers. Leukocytes were stimulated with Leukocyte Activation Cocktail (BD Pharmingen, San Diego, CA) at 37 °C for 4 h prior to intracellular staining using the manufacturer’s staining protocol. Anti-human monoclonal antibodies (mAbs) against PE-CF594-CD3, APC-CD4, V450-CD8, FITC-IFN-γ, PE-IL-4, APC-IL-17A, and APC-IL-10 with corresponding isotype-matched controls were purchased from BD Biosciences (San Jose, CA, USA). Data were acquired using a Gallios instrument (Beckman Coulter, Brea, CA) and analyzed with FlowJo software (Ashland, OR).

### Clinical and serological parameters

Upon recruitment, patient serum was tested for HBsAb, HBeAb, HBeAg using commercial kits (Abbott Laboratories, North Chicago, IL). Quantitative HBsAg (qHBsAg) (dynamic range from 0.05 to 52,000 IU/ml) and HBsAb levels were measured with the Elecsys HBsAg II Quant reagent kits (Roche Diagnostics, Indianapolis, IN) according to the manufacturer’s instructions. Serum HBV DNA level was measured by Roche COBAS Ampliprep/COBAS TaqMan HBV Test v2.0 (dynamic range from 20 to 1.7E + 08 IU/mL, Roche Molecular Diagnostics, Branchburg, NJ). Level of fibrosis was defined by liver stiffness measurement (Fibroscan, Echosens, Paris, France). Genotyping of HBV was carried out by polymerase chain reaction-restriction fragment length polymorphism of the surface gene of HBV as previously described [11, 30]. Briefly, the extracted DNA was amplified for the fragment of the HBV genome between nucleotide positions 256 and 796. The polymerase chain reaction products were subsequently treated with restriction enzymes. After incubation, the samples were run on a 3% agarose gel and stained by ethidium bromide. Six genotypes (A-F) of HBV were identified by the restriction patterns of DNA fragments. Unclassified genotype was defined as an unpredictable or atypical restriction pattern.

### Statistical analysis

We compared two patient groups using the Mann-Whitney test for continuous variables and the χ2 test for categorical variables. We explored the association between two continuous variables using a linear regression model, Pearson correlation or Spearman correlation. All other statistical tests were performed using R software version 3.2.2. Statistical significance was set to 0.05.

## Results

### Peripheral blood T cell subsets and cytokine profiles in different disease phases of CHB patients

**Fig. 1:**
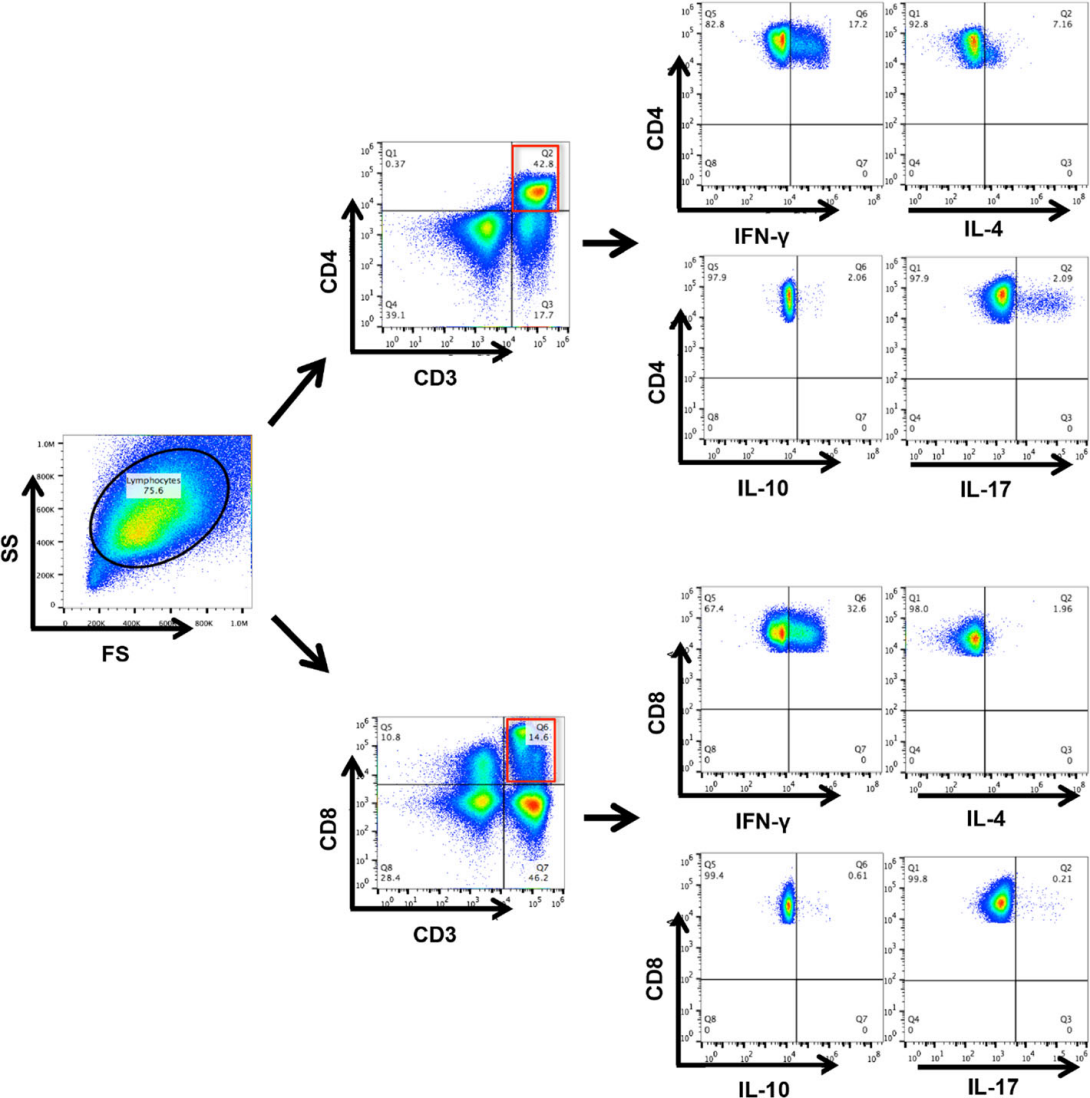
Gating strategy for IL-4, IL-10, IFN-γ, IL-17 produced by CD4^+^ and CD8^+^ T cells. T cells were derived from total live PBMCs gated by forward and side scatter followed by single-cell gating using width and height parameters. CD4^+^ and CD8^+^ T cells were defined by the co-expression of CD3 and CD4 or CD8. The above cells were shown in the red boxes as indicated. Percentage of IL-4, IL-10, IFN-γ, IL-17 produced by CD4^+^ and CD8^+^ T cells were further calculated according to the fluorescence of each cytokine antibody

**Fig. 2:**
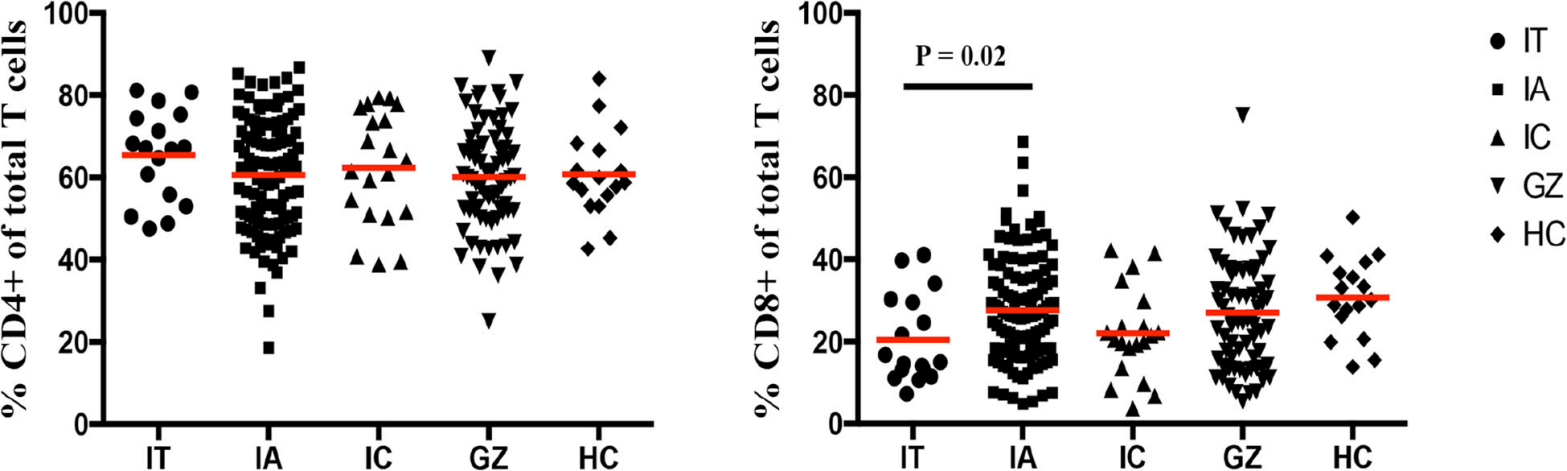
Frequency of CD4^+^ and CD8^+^ T cells in IT (*n* = 17), IA (*n* = 120), IC (*n* = 20) and GZ (*n* = 72) phases of naïve CHB patients, and of healthy control (*n* **=** 17**).** The levels were compared among these patients in different phases, and those of healthy controls. (IT, immune tolerant; IA, immune active; IC, inactive carrier; GZ, gray zones)

To investigate T cell immunity in the current untreated patient cohort, we characterized the frequencies of T cell subsets and their secreted cytokines in 229 CHB patients in different phases of the disease. Gating strategy of flow cytometry for cytokines produced by CD4^+^ and CD8^+^ T cells is shown in Fig. 1. The clinical features of the patient cohort studied are shown in Table 1. We first analysed proportions of CD4^+^ and CD8^+^ T cells and compared these T cell profiles among different patient groups. No statistically significant differences in the distribution of CD4^+^ T cells were observed among the IA, IT, IC and GZ groups or healthy control (Fig. 2). In contrast, the frequency of CD8^+^ T cells was significantly increased in patients in the IA phase compared to those in the IT phase (*P* = 0.02), suggesting higher cytotoxic activity in patients with increased liver inflammation.

**Fig. 3:**
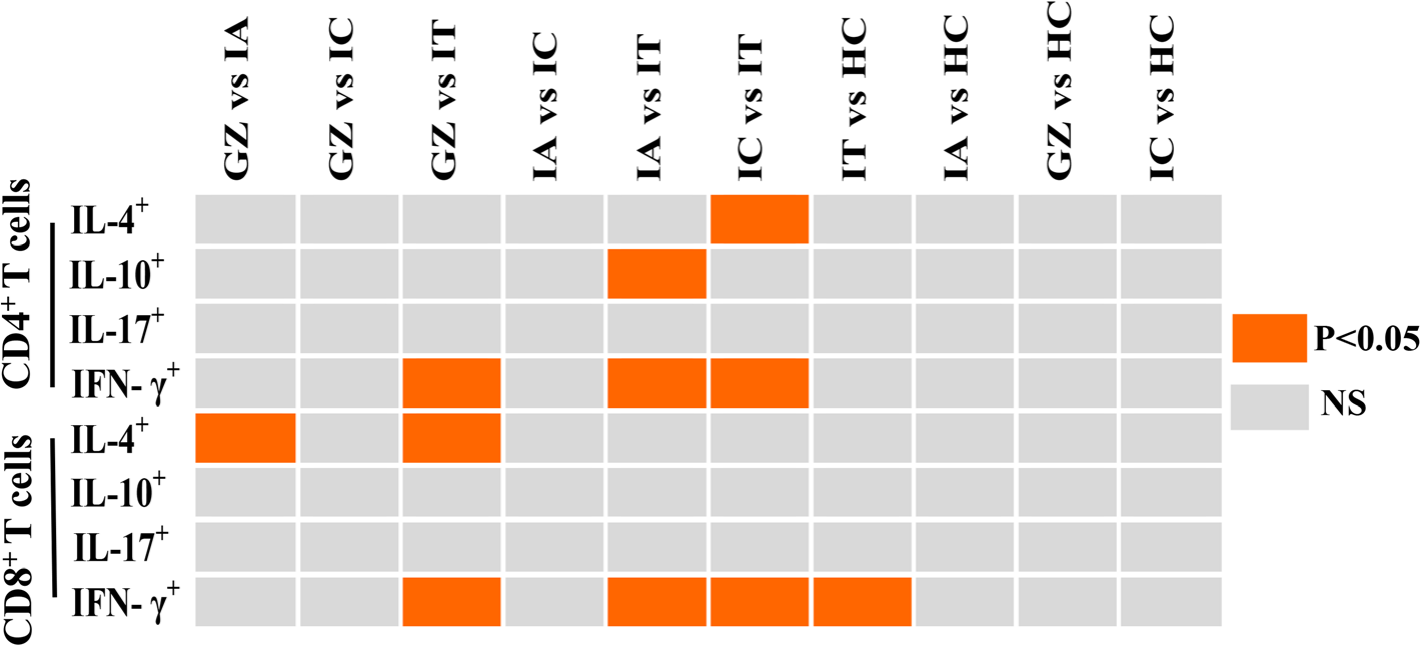
Comparison of cytokines IL-4, IL-10, IFN-γ, IL-17 produced by CD4^+^ and CD8^+^ T cells among naïve CHB patients in IT, IA, IC, GZ phases and healthy control. Differences between multiple groups had been evaluated by Wilcoxon rank sum test. *P* <  0.05 was colored in red. NS, not significant. (IT, immune tolerant; IA, immune active; IC, inactive carrier; GZ, gray zones)

The frequencies of T cell subsets producing effector/inflammatory cytokines (IFN-γ and IL-17), T helper cytokine (IL-4) and immunomodulatory cytokine (IL-10) were also analyzed. Compared to the levels observed in other patient groups, a significant decrease in IFN-γ level was detected in both CD4^+^ and CD8^+^ T cells from IT patients. Moreover, discrepancies in the frequencies of IL-4 and IL-10 by T-cell subset in patients at different disease phases were also observed. Patients in the IC and IA phases exhibited significantly higher levels of IL-4 and IL-10 produced by CD4^+^ T cells, respectively, in comparison to the levels observed in IT patients. An increase in IL-4 from CD8^+^ T cells was also observed in GZ patients when compared to patients of the IA and IT phases (Fig. 3). No variation in IL-17 expression was found for any T cell subset among the patient groups.

Taken together, these data demonstrate that the lowest frequency of T cells and their cytokine functions were in IT patients. However, no divergent behaviours of T cell and cytokine profiles, as defined by three clinical-virological parameters (ALT, HBV DNA, HBeAg), were observed among other patient groups. We therefore included patient demographics, HBV markers, ALT, and liver fibroscan values to explore correlations between cellular immunity and HBV status using linear regression.

### Correlation of T cell-producing Th2 cytokine and immunomodulatory cytokine to viral factors

A linear regression model was used to examine associations of Th2 cytokine (IL-4) and immunomodulatory cytokine IL-10 to clinical-virological factors in this untreated CHB cohort. Univariate analysis revealed that higher levels of HBsAg, HBeAg and HBV DNA were significantly associated with decreased frequencies of CD4^+^ T cells IL-4^+^ (*P* = 0.005, 0.012, and 0.01 for HBsAg, HBeAg and HBV DNA, respectively). Similar results were found regarding the correlation between above-mentioned HBV factors and CD8^+^ T cells IL4^+^ (*P* = 0.004, 0.005, and 0.000 for HBsAg, HBeAg and HBV DNA, respectively). Moreover, increased HBeAb titers and the presence of PC mutation were correlated with higher IL-4 secreted by CD4^+^ and CD8^+^ T cells, respectively (*P* = 0.026, 0.037, for the relationship between HBeAb and CD4^+^ T cells IL-4^+^, and between PC mutation and CD8^+^ T cells IL-4^+^, respectively). Multivariate analysis after adjusting for other confounding factors showed that HBsAg and HBV DNA remained significantly negatively correlated to IL-4 secreted by CD4^+^ and CD8^+^ T cells, respectively (*P* = 0.005, 0.000, for the relationship between HBsAg and CD4^+^ T cells IL-4^+^, and between HBV DNA and CD8^+^ T cells IL-4^+^, respectively, Table 2).

**Table 2:** Factors associated with CD4^+^ and CD8^+^ T cells secreting IL-4 and IL-10 in the univariate and multivariate linear regression analysis

Variable	CD4^+^ T cells IL-4^+^				CD4^+^ T cells IL-10^+^				CD8^+^ T cells IL-4^+^				CD8^+^ T cells IL-10^+^			
	Univariate (raw effects)		Multivariate (adjusted effects)		Univariate (raw effects)		Multivariate (adjusted effects)		Univariate (raw effects)		Multivariate (adjusted effects)		Univariate (raw effects)	Multivariate (adjusted effects)		
	B (95% CI)	*P*	Adjusted B (95%CI)	Adjusted *P*	B (95% CI)	*P*	Adjusted B (95%CI)	Adjusted P	B (95% CI)	*P*	Adjusted B (95% CI)	Adjusted P	B (95% CI)	*P*	Adjusted B (95% CI)	Adjusted *P*
Age (≥ 25 vs < 25 years)	0.18(− 0.11,0.48)	0.226			− 0.31(− 0.87,0.24)	0.266			0.05(− 0.11,0.2)	0.559			−0.21(− 0.48,0.05)	0.118		
Gender (Male vs Female)	0.22(− 0.03,0.48)	0.087			0.02(− 0.47,0.51)	0.935			0.02(− 0.11,0.16)	0.723			−0.13(− 0.36,0.1)	0.259		
ALT (< 2 ULN is the reference group)																
≥ 2 & < 5 ULN	−0.21(−0.52,0.09)	0.171			−0.37(− 0.95,0.21)	0.205			−0.23(− 0.38,-0.08)	**0.004**			−0.2(− 0.47,0.08)	0.158		
≥ 5ULN	−0.19(− 0.6,0.22)	0.363			−0.27(−1.04,0.49)	0.480			−0.29(− 0.49,-0.09)	**0.005**			−0.18(− 0.54,0.19)	0.339		
Fibroscan	0.06(−0.32,0.43)	0.767			−0.2(− 0.91,0.5)	0.570			0.04(−0.16,0.23)	0.714			−0.14(− 0.47,0.2)	0.426		
Log HBV DNA	−0.09(− 0.15,-0.02)	**0.010**			−0.1(− 0.23,0.02)	0.094			−0.06(− 0.1,-0.03)	**0.000**	− 0.06(− 0.1,− 0.03)	**0.000**	-0.03(− 0.09,0.03)	0.315		
Log HBsAg	− 0.2(− 0.34,-0.06)	**0.005**	−0.2(− 0.34,-0.06)	**0.005**	0.87 (0.18,1.56)	**0.014**	−0.28(− 0.54,-0.03)	**0.030**	−0.1(− 0.17,-0.03)	**0.004**			−0.09(− 0.22,0.03)	0.153		
HBeAg(Positive)	−0.32(− 0.58,-0.07)	**0.012**			−0.35(− 0.83,0.13)	0.154			−0.19(− 0.31,-0.06)	**0.005**			−0.05(− 0.28,0.19)	0.693		
HBeAb(Positive)	0.29 (0.03,0.54)	**0.026**			0.28(−0.2,0.76)	0.244			0.11(−0.02,0.25)	0.084			0.02(−0.21,0.25)	0.884		
HBsAb(Positive)	0.18(−0.2,0.55)	0.349			0.87 (0.18,1.56)	**0.014**	0.87 (0.19,1.54)	**0.013**	−0.05(− 0.25,0.14)	0.593			0.37 (0.03,0.7)	**0.032**	0.37 (0.03,0.7)	**0.032**
Genotype (Genotype C is the reference group)																
B	−0.18(−0.47,0.11)	0.214			0.38(−0.17,0.92)	0.174			−0.04(− 0.19,0.11)	0.637			0.23(−0.03,0.49)	0.078		
Other	−0.24(− 0.66,0.18)	0.262			0.18(−0.6,0.97)	0.643			0(−0.22,0.21)	0.969			0.14(−0.23,0.52)	0.450		
PC mutation (Positive)	0.17(−0.09,0.44)	0.201			0.46(−0.03,0.95)	0.068			0.1(−0.03,0.24)	0.135			0.09(−0.15,0.33)	0.451		
BCP mutation (Positive)	0.1(−0.17,0.38)	0.455			−0.25(− 0.76,0.26)	0.340			0.09(− 0.05,0.22)	0.230			−0.13(− 0.37,0.11)	0.296		
PC and BCP mutations (Positive)	0(−0.38,0.37)	0.988			−0.16(− 0.87,0.55)	0.655			0.2 (0.01,0.39)	**0.037**			−0.03(− 0.37,0.31)	0.857		

Significant values are shown in boldface. B: Unstandardized Coefficients. HBV genotype: Other included C + D, B + D, B + C, D, and not detected

ALT, alanine aminotransferase; HBeAb, antibody to HBV e antigen; HBeAg, HBV e antigen; HBsAb: antibody to hepatitis B surface antigen; HBsAg, hepatitis B surface antigen; PC: precore; BCP: basal core promoter

Similarly, linear regression was used to examine the association between IL-10 by T cell subsets and virological factors. Increased HBsAg levels were significantly associated with lower expression of CD4^+^ T cells IL-10^+^, as indicated by multivariate analysis (*P* = 0.033). Conversely, IL-10 produced by both T cell subsets exhibited significantly positive correlations with HBsAb titers (*P* = 0.014, 0.032, for the relationship between HBsAb and CD4^+^ T cells IL-10^+^, and between HBsAb and CD8^+^ T cells IL-10^+^, respectively, Table 2).

Taken together, these findings indicated an association of Th2 cytokine and immunomodulatory cytokine to HBV protein status, viral replication, and mutation.

### Effector cytokine responses associated with clinical virology and liver inflammation

The linear regression model demonstrated that older age, higher ALT level, and lower HBsAg titer were significantly correlated with increased CD4^+^ T cells IFN-γ^+^ (*P* = 0.003, 0.000 and 0.029, for the relationship between age, ALT or HBsAg, respectively, and CD4^+^ T cells IFN-γ^+^). CD8^+^ T cells IFN-γ^+^ was also observed to be correlated with age and levels of ALT and HBsAg (*P* = 0.004, 0.000 and 0.004, for the relationship between age, ALT or HBsAg, respectively, and CD8^+^ T cells IFN-γ^+^). Similar results were observed after adjusting for other confounding factors (Table 3). There was also a statistically significant positive relationship between the frequency of CD4^+^ T cells IFN-γ^+^ and the fibroscan value (*P* = 0.005). However, multivariate analysis indicated a lack of a statistically significant correlation.

**Table 3:** Factors associated with CD4^+^ and CD8^+^ T cells secreting IFN-γ and IL-17 in the univariate and multivariate linear regression analysis

	CD4^+^ T cells IFN-γ^+^				CD4^+^ T cells IL-17^+^				CD8^+^ T cells IFN-γ^+^				CD8^+^ T cells IL-17^+^			
Variable	Univariate (raw effects)		Multivariate (adjusted effects)		Univariate (raw effects)		Multivariate (adjusted effects)		Univariate (raw effects)		Multivariate (adjusted effects)		Univariate (raw effects)		Multivariate (adjusted effects	
	B (95% CI)	*P*	Adjusted B (95%CI)	Adjusted *P*	B (95% CI)	*P*	Adjusted B (95%CI)	Adjusted P	B (95% CI)	*P*	Adjusted B (95% CI)	Adjusted P	B (95% CI)	*P*	Adjusted B (95% CI)	Adjusted *P*
Age (≥ 25 vs < 25 years)	5.09 (1.75,8.43)	**0.003**	3.66 (0.61,6.72)	**0.019**	−0.12(− 0.51,0.26)	0.521			9.5 (3.07,15.94)	**0.004**	6.85 (0.79,12.92)	**0.027**	−0.03(− 0.15,0.1)	0.659		
Gender (Male vs Female)	1.08(−1.93,4.08)	0.480			0.21(−0.12,0.54)	0.206			2.42(−3.35,8.18)	0.408			0.07(− 0.04,0.18)	0.192		
ALT (< 2 ULN is the reference group)																
≥ 2 & < 5 ULN	7.18 (3.9,10.45)	**0.000**	7.42 (4.3,10.54)	**0.000**	0.06(−0.34,0.46)	0.756			11.88 (5.34,18.43)	**0.000**	12.57 (6.39,18.75)	**0.000**	−0.12(− 0.24,0.01)	0.067		
≥ 5ULN	6.25 (1.9,10.61)	**0.005**	6.48 (2.36,10.61)	**0.002**	0.16(−0.37,0.69)	0.548			2.9(−5.8,11.6)	0.511	3.54(−4.63,11.71)	0.392	−0.11(− 0.28,0.06)	0.190		
Fibroscan	6.08 (1.85,10.32)	**0.005**			0.04(−0.45,0.52)	0.877			6.15(−2.19,14.49)	0.147			−0.12(− 0.28,0.03)	0.114		
Log HBV DNA	−0.47(−1.24,0.29)	0.225			−0.01(− 0.09,0.08)	0.851			−1.01(−2.48,0.45)	0.174			−0.02(− 0.05,0)	0.097		
Log HBsAg	−1.81(−3.42,-0.19)	**0.029**	−2.01(−3.47,-0.55)	**0.007**	0.02(−0.17,0.2)	0.866			−4.52(−7.57,-1.46)	**0.004**	−4.63(−7.53,-1.74)	**0.002**	−0.02(− 0.08,0.04)	0.427		
HBeAg(Positive)	−1.93(−4.9,1.04)	0.200			0.1(−0.23,0.43)	0.543			−5.32(−10.98,0.34)	0.065			−0.06(− 0.16,0.05)	0.281		
HBeAb(Positive)	3.21 (0.3,6.13)	**0.031**			−0.07(−0.4,0.26)	0.679			6.25 (0.66,11.85)	**0.029**			0.05(−0.06,0.15)	0.371		
HBsAb(Positive)	2.98(−1.36,7.33)	0.177			0.13(−0.35,0.62)	0.587			5.92(−2.43,14.26)	0.163			−0.09(− 0.24,0.07)	0.283		
Genotype (Genotype C is the reference group)																
B	−1.93(−5.26,1.4)	0.253			−0.28(−0.65,0.09)	0.140			−4.65(−11.08,1.78)	0.155			−0.01(− 0.13,0.11)	0.889		
Other	−5.33(−10.14,-0.52)	**0.030**			−0.02(− 0.56,0.52)	0.946			−8.54(−17.82,0.74)	0.071			0.02(−0.16,0.19)	0.857		
PC mutation (Positive)	3.6 (0.58,6.62)	**0.020**			−0.28(−0.62,0.06)	0.106			5.22(−0.64,11.07)	0.080			0.01(−0.1,0.12)	0.834		
BCP mutation (Positive)	0.7(−2.46,3.87)	0.661			0.03(−0.32,0.38)	0.859			4.46(−1.57,10.49)	0.146			−0.03(− 0.15,0.08)	0.561		
PC and BCP mutations (Positive)	−0.07(−4.46,4.31)	0.974			−0.51(− 0.98,-0.03)	**0.037**	− 0.51(− 0.98,-0.03)	**0.037**	2.02(−6.39,10.43)	0.635			−0.13(− 0.28,0.03)	0.107		

Significant values are shown in boldface.B: Unstandardized Coefficients. HBV genotype: Other included C + D, B + D, B + C, D, and not detected

ALT, alanine aminotransferase; HBeAb, antibody to HBV e antigen; HBeAg, HBV e antigen; HBsAb: antibody to hepatitis B surface antigen; HBsAg, hepatitis B surface antigen; PC: precore; BCP: basal core promoter

In addition to IFN-γ, data from multivariate analysis demonstrated a correlation between reduced levels of cytokine IL-17 and the presence of PC and BCP mutation of HBV genome (*P* = 0.037, Table 3). However, this phenomenon was only observed for the relationship between PC mutation and CD4^+^ T cells IL-17^+^ and not CD8^+^ T cells IL-17^+^.

### Associations among immunological responses

**Fig. 4:**
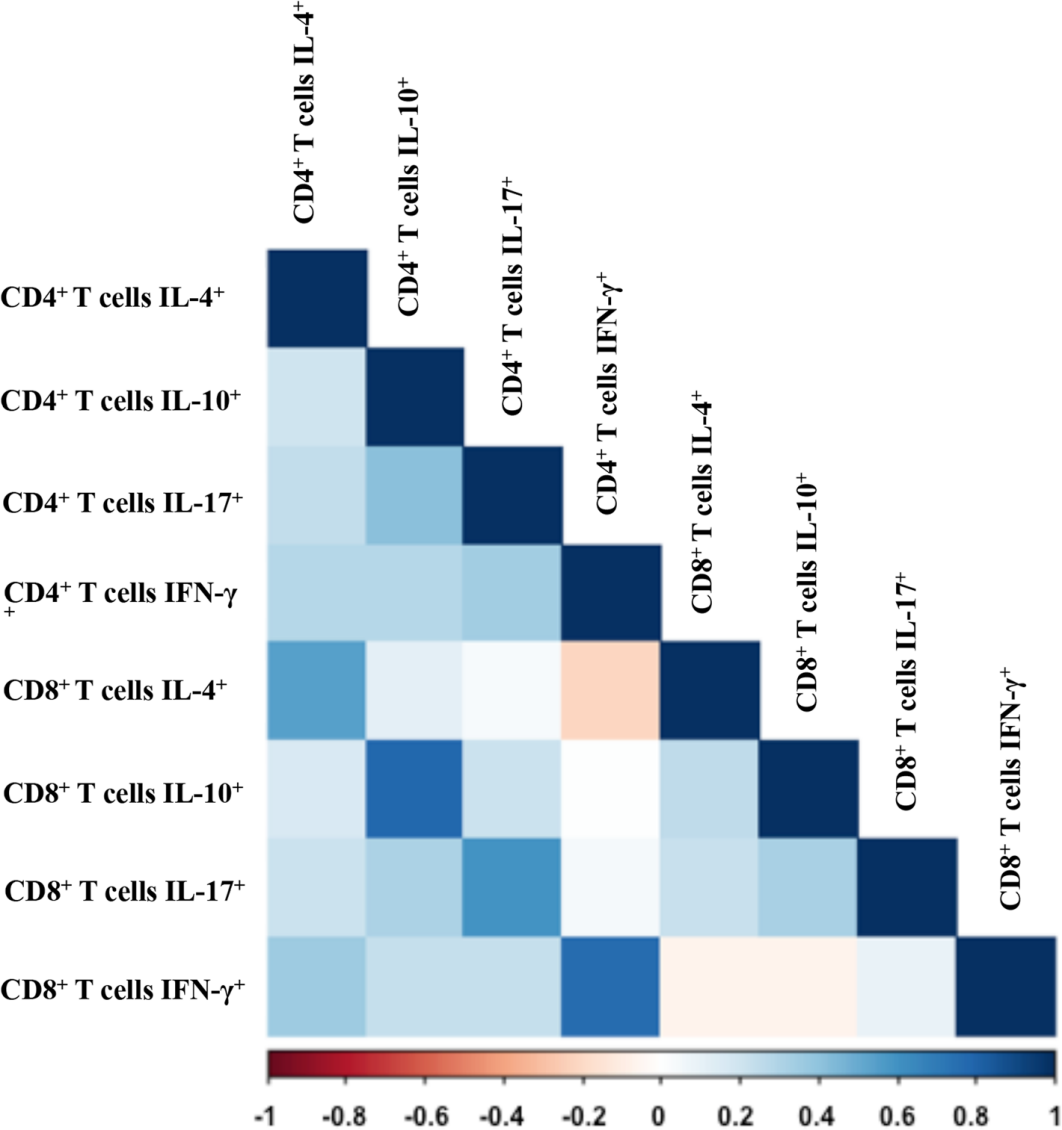
Correlations among IL-4, IL-10, IFN-γ and IL-17 produced by CD4^+^ and CD8^+^ T cells measured by Spearman correlation. *P* <  0.05 was colored, and pseudocolours indicate correlation levels from negative (− 1) to positive (1), ranging from a weak (white) to strong (red or blue) association strength

Because individual T cell cytokines were found to be correlated with one or more clinical-virological parameters in this untreated CHB cohort, we further investigated associations among frequencies of IL-4, IL-10, IL-17, and IFN-γ by T cell subsets. As expected, there was a significant association between CD4^+^ T cells IL-4^+^ and CD8^+^ T cells IL-4^+^ (r = 0.54, *P* <  0.05). Similar findings were observed for relationships between CD4^+^ T cells IL-10^+^ and CD8^+^ T cells IL-10^+^ (r = 0.78), and CD4^+^ T cells IL-17^+^ and CD8^+^ T cells IL-17^+^ (r = 0.6), and CD4^+^ T cells IFN-γ^+^ and CD8^+^ T cells IFN-γ^+^ (r = 0.77), respectively. Notably, although statistically significant associations were observed among these cytokines by CD4^+^ or CD8^+^ T cells, the correlation coefficients were small, suggesting weak autocorrelations among these cytokines, which indicated that IL-4, IL-10, IL-17, and IFN-γ were nearly independently correlated with HBV factors (Fig. 4).

## Discussion

This study demonstrates associations between several effector or helper cytokines produced by T cell subsets and the levels of HBV markers and liver inflammation in 229 treatment-naïve CHB patients. In particular, the frequencies of CD4^+^ T cells IL-4^+^ or IL-10^+^ were strongly associated with HBsAg or HBsAb. Levels of IFN-γ secreted by CD4^+^ and CD8^+^ T cells were also significantly associated with older age and increased ALT and HBsAg. In addition, the frequency of CD4^+^ T cells IL-17^+^ was significantly higher in patients with PC mutation than those with wild-type PC sequences.

In the natural course of HBV infection, HBsAg loss occurs at an annual rate of 0.5–2.3% [31–33]. Quantitative HBsAg levels can predict the seroclearance of HBsAg in HBeAg seroconverters or HBeAg-seronegative patients with low viral loads [34]. For patients with low HBV DNA, the risk of HCC development is considered in an HBsAg titer-dependent manner [35]. By Integrating the characteristics of cellular immunity and the virus, we observed significant associations for both IL-4 and IFN-γ with the HBsAg levels, despite the counteracted characteristics between them. IL-4 is a multifunctional pleiotropic cytokine that plays important roles as a mediator and modulator of inflammatory immunity [36]. Inconsistent with previous reports that IL-4 is positively correlated with serum viral load and ALT to exert its suppressive effect on T helper cytokine function by promoting neutrophil survival and hepatitis [37], the observed contrasting significant correlations of IL-4 with HBeAg and HBeAb in the current study primarily demonstrates the essential regulation of Th2 cell response during HBeAg seroconversion. These divergent signatures of IL-4 in CHB may be due to gene polymorphisms [36], and it might need further study to investigate the relationship between gene polymorphisms and HBeAg seroconversion or antiviral efficacy. In addition, the significantly positive correlations between IFN-γ and age or ALT represent the inflammatory features of IFN-γ [38–40].

HBV mutations might play a pivotal role in liver disease aggravation and virus replication. PC mutations (G1896A) and BCP double mutations (T1762/A1764) are important changes that alter expression of HBeAg [12], which is important for establishing viral persistence. Tong et al. reported that 42% of cirrhosis patients and 45% of HCC patients have the G1896A PC mutation, with the latter indicating a high risk of developing HCC [41]. Despite numerous related studies, the significance of this mutation remains obscure. According to our data, 32.5, 27.2, and 11.3% of the patients developed BCP, PC and combined BCP and PC mutations, respectively. Only combined BCP and PC mutations was found to be negatively associated with the frequency of CD4^+^ T cells IL-17^+^. IL-17 can be contributed to the immune activation and disease aggravation in patients with chronic HBV infection, which might be related to viral persistence caused by HBV mutation, but there was few study on the relationship between IL-17 and HBV mutations, and it needs further studies. Within the feedback loop of Th1 and Th2 cytokines, IL-4 mediated Th2 cells differentiation and stability, thereby inhibiting cytokine production by Th1 cells [42, 43]. The associations observed in current study cohort confirmed a significantly positive and negative correlation between the frequency of CD4^+^ T cells IL-17^+^ and presence of PC mutation as well as between the frequency of CD4^+^ T cells IL-10^+^ and HBsAb level, respectively. High IL-10 production was shown to be associated with greater ALT levels during the immune-clearance phase and greater rate of decrease in HBsAg titer, which were both key elements before spontaneous HBsAg seroconversion [44]. Opposite significant correlations of IL-10 between HBsAg and HBsAb in current study mainly showed the essential regulation response of B cell in the process for fighting against infections. We failed to identify significant associations between IL-10 and IL-17 secreted by CD4^+^ T cells in this treatment-naïve HBV cohort, suggesting that these two cytokines independently influence HBV antigen expression. These cells may interact during immune protection and immune inflammatory events throughout the disease phases of CHB.

Genotype B is noted to associate with less severe liver disease and early spontaneous HBeAg seroconversion than non-genotype B HBV in both adults and children in Taiwan [11, 45]. IL-10 can inhibit formation of pro-inflammatory cytokines and correlated to anti-HBs response and HBeAg seroconversion [46, 47]. In our study, multivariate analysis demonstrated that IL-10 produced by CD8^+^ T cells was nearly positive correlated with genotype B, not other genotypes, indicating that IL-10 produced by CD8^+^ T cells might play an important role in the pathogenesis of the less sever liver disease of genotype B, and this need further study.

In conclusion, this study reveals that several components of the immune system are correlated with HBV factors that influence an inflammatory process during CHB. Of particular relevance are the significant associations between the frequency of CD4^+^ T cells IL-4^+^ and quantitative HBsAg level, and between the frequency of CD4^+^ T cells IL-17^+^ and the presence of PC mutation. The associations among the cytokines were independent from each other, even though significant relationships with one or more HBV markers were found. The limitation of this study was that data were gathered at a single point in time for each subject and analyzed by the linear regression model to identify the associations among clinical laboratory characteristics, virological information, and immunological parameters. Nonetheless, the results of this study highlight the importance of the host-virus relationship based on T cell effector or helper cytokines during immunity and immunopathology in CHB.

## Conclusions

Several components of the immune system are correlated to HBV factors that influence an inflammatory process during CHB. Of particular relevance are the significant associations of between CD4^+^ T cells IL-4^+^ and qHBsAg, and between CD4^+^ T cells IL-17^+^ and the presence of PC mutation.

## Abbreviations

ALT: alanine aminotransferase
BCP: basal core promoter
CHB: chronic hepatitis B
GLB: globulin
GZ: gray zones
HBeAb: antibody to HBV e antigen
HBeAg: HBV e antigen
HBsAb: antibody to hepatitis B surface antigen
HBsAg: hepatitis B surface antigen
HBV: hepatitis B virus
HCC: hepatocellular carcinoma
IA: immune active
IC: inactive carrier
IFN-γ: interferon-γ
IL: interleukin
IT: immune tolerant
PBMCs: peripheral blood mononuclear cells
PC: precore
Th: T helper
